# Using Breast Cancer Risk Associated Polymorphisms to Identify Women for Breast Cancer Chemoprevention

**DOI:** 10.1371/journal.pone.0168601

**Published:** 2017-01-20

**Authors:** Elad Ziv, Jeffrey A. Tice, Brian Sprague, Celine M. Vachon, Steven R. Cummings, Karla Kerlikowske

**Affiliations:** 1 Department of Medicine, University of California, San Francisco, California, United States of America; 2 Helen Diller Family Comprehensive Cancer Center, University of California, San Francisco, California, United States of America; 3 Institute for Human Genetics, University of California, San Francisco, California, United States of America; 4 Department of Epidemiology and Biostatistics, University of California, San Francisco, California, United States of America; 5 Department of Surgery and University of Vermont Cancer Center, University of Vermont, Burlington, Vermont, United States of America; 6 Department of Health Sciences Research, Division of Epidemiology, Mayo Clinic College of Medicine, Rochester, Minnesota, United States of America; 7 San Francisco Coordinating Center, California Pacific Medical Center Research Institute, San Francisco, California, United States of America; 8 General Internal Medicine Section, Department of Veterans Affairs, University of California, San Francisco, California, United States of America; National Health Research Institutes, TAIWAN

## Abstract

**Background:**

Breast cancer can be prevented with selective estrogen receptor modifiers (SERMs) and aromatase inhibitors (AIs). The US Preventive Services Task Force recommends that women with a 5-year breast cancer risk ≥3% consider chemoprevention for breast cancer. More than 70 single nucleotide polymorphisms (SNPs) have been associated with breast cancer. We sought to determine how to best integrate risk information from SNPs with other risk factors to risk stratify women for chemoprevention.

**Methods:**

We used the risk distribution among women ages 35–69 estimated by the Breast Cancer Surveillance Consortium (BCSC) risk model. We modeled the effect of adding 70 SNPs to the BCSC model and examined how this would affect how many women are reclassified above and below the threshold for chemoprevention.

**Results:**

We found that most of the benefit of SNP testing a population is achieved by testing a modest fraction of the population. For example, if women with a 5-year BCSC risk of >2.0% are tested (~21% of all women), ~75% of the benefit of testing all women (shifting women above or below 3% 5-year risk) would be derived. If women with a 5-year risk of >1.5% are tested (~36% of all women), ~90% of the benefit of testing all women would be derived.

**Conclusion:**

SNP testing is effective for reclassification of women for chemoprevention, but is unlikely to reclassify women with <1.5% 5-year risk. These results can be used to implement an efficient two-step testing approach to identify high risk women who may benefit from chemoprevention.

## Introduction

Breast cancer risk can be reduced in women with the use of selective estrogen receptor modifiers (SERMs) such as tamoxifen[1] and raloxifene [2]or with the use of aromatase inhibitors (AIs)[3]. However, these drugs can also cause significant side effects and increase the risk of adverse events such as venous thromboembolic disease in the case of SERMs or osteoporosis in the case of AIs. Thus, they are only recommended for women at elevated risk of breast cancer and, currently, they are not widely used in the prevention setting[4]. Recently, the US Preventive Services Task Force (USPSTF) issued a recommendation that women at ≥3% 5 year risk of breast cancer consider preventive therapy for breast cancer[5].

To target these preventive medications to the women most likely to benefit, physicians need to be able to estimate a woman’s risk for breast cancer[6]. A variety of models exist to risk stratify women for breast cancer including the Gail model[7], the Tyrer-Cuzick model[8] and the Breast Cancer Surveillance Consortium (BSCC) model[9]. These models combine family history, reproductive risk factors, history of benign breast disease and mammographic density to risk stratify women.

Genome wide association studies have identified >80 genetic variants or single nucleotide polymorphisms (SNPs) associated with breast cancer risk[10–17]. Each variant only modestly alters breast cancer risk; however, together they add substantial risk information to current risk predictors[18]. Thus, when added to traditional risk models, a combination of genetic risk factors could be used to enhance risk stratification.

As with any clinical test, genetic risk factors should only be ordered if the result is likely to affect clinical management. In the context of chemoprevention, genetic risk factors would need to either increase the risk of breast cancer above the threshold to initiate therapy, or decrease the risk substantially so that women who are above the threshold for therapy are reclassified as being below the threshold. Here we use a modeling approach to consider different strategies for joint non-genetic and genetic risk stratification for breast cancer. In particular, we simulate the combination of risk in a population of women being screened for breast cancer, starting with their BCSC model estimated risk and adding genetic information on 70 risk SNPs.

## Materials and Methods

### Data

We used data from the BCSC to generate the risk distributions for women, including all Caucasian women between the ages of 35–69 years who received screening mammograms and had risk factors available to calculate a BCSC 5-year risk estimate. The BCSC risk model includes age, race/ethnicity, family history of breast cancer, mammographic density and history of breast biopsy[9]. We used an upper threshold of age 69 since the benefit of tamoxifen for prevention takes several years to realize and therefore, we assumed that it is more likely to be initiated in younger women where long term benefits would be more likely to outweigh harms. We did not model the risk of women from other racial/ethnic groups since the odds ratios with the SNPs are not well-described in those populations. For each woman a 5-year risk was calculated using the BCSC model. We then categorized women in increasing categories of risk, with each category representing a 0.2% increase in 5- year risk (i.e. category 1: 0–0.2%, category 2: 0.21–0.4%,….). We calculated the fraction of women in each risk category(S1 Table). The calculation was performed for all non-Hispanic White women in the BCSC database (N = 796,294), since the odds ratios for the risk SNPs are best characterized in that population. Women estimated to have >6% 5-year risk were grouped as one category.

We also calculated the risk categories for the following subsets of women: ages 35–49 and women 50–69 years; with and without family history of breast cancer; women with mammographic density considered almost entirely fatty, scattered fibroglandular densities, heterogeneously dense, and extremely dense.

### SNP Selection

We selected SNPs (S2 Table) discovered by GWAS[10–12, 14–17, 19–26]. We used the allele frequencies and odds ratios for breast cancer from Caucasian populations for each SNP. To eliminate the possibility that the SNPs are giving redundant risk information, we calculated the linkage disequilibrium (LD) between SNPs that are in the same locus using LDlink[27] and dropped SNPs in linkage disequilibrium. We used a conservative threshold (R^2^>0.2 or D’>0.2) to minimize the possibility of bias in our risk calculation. To select SNPs among any set of SNP in LD we selected the SNP with the highest product between allele frequency and odds ratio. After removing SNPs in LD, we were left with 70 SNPs (S2 Table). The vast majority of these SNPs have previously been validated empirically to predict independently of each other[28, 29] and to be independent of the BCSC risk model[18].

### Modeling Genetic Risk

#### Simulating genotypes

For a screening population we used the published allele frequencies in Caucasian populations. We then simulated genotypes based on the assumption that each risk allele is in Hardy Weinberg equilibrium and that the genotypes are inherited independently.

To simulate the genotypes by age and other categories, we assumed independence of the genotypes and these risk factors except for family history where we explicitly modeled the expected effect of family history on the distribution of family history on genotypes (S1 Table). We simulated 10,000 individuals for each scenario.

#### Outline of the polygenic risk calculation score

Our approach to calculating the polygenic risk score is based on a Bayesian approach. Each woman starts with a prior probability (based on a risk model without SNPs such as the BCSC). For each genotype at each SNP we calculate the probability of having that genotype among patients with breast cancer and use that to update the probability of having breast cancer in a particular woman.

#### Calculating polygenic risk score

The probability of breast cancer for women with a particular genotype, Gi, can be calculated using Bayes’ Theorem as shown in[30]:

$$P\left(D^{+}|G_{i}\right)=\;\frac{P\left(D^{+}\right)P\left(G_{i}|D^{+}\right)}{P\left(G_{i}\right)}$$ (1)

Where P(D^+^) is the prior probability (prior to genotyping) of developing breast cancer, P(G_i_) is the frequency of the particular G_i_ in the population and P(G_i_|D^+^) is the probability of having a genotype G_i_ among breast cancer cases. A related, commonly used measure of the effect of a clinical test, the likelihood ratio (LR) associated with the genotype, can be represented as[31, 32]:

$$LR_{Gi}=\;\frac{P(G_{i}|D^{+})}{P(G_{i}|D^{-})}$$ (2)

For any genotype G_i_ genotypes then the probability of having the genotype G_i_ among patients with the disease state (D^+^) can be approximated by

$$P\left(G_{i}|D^{+}\right)=\frac{P\left(G_{i}\right)\gamma_{i}}{\sum_{i=1}^{n}P\left(G_{i}\right)\gamma_{i}}$$ (3)

given a disease with relatively low prevalence (<10%). The probability of having genotype Gi among people without disease (D^−^) is given by

$$P\left(G_{i}|D^{-}\right)=P\left(G_{i}\right)-\frac{P\left(G_{i}\right)\gamma_{i}K}{\left(1-K\right)\sum_{i=1}^{n}P\left(G_{i}\right)\gamma_{i}}$$ (4)

where P(Gi) is the probability of the genotype in the general population, γ_i_ is the relative risk associated with that genotype and K is the prevalence of the disease.

Assuming all of the SNPs are inherited independently (in linkage equilibrium) and that there are no interactions between them, then the LR for each multi-SNP genotype, G_i_ is the product of the likelihood ratios for the genotype, g_i_ of each of the n SNPs.

$$LR_{Gi}=\;\prod_{i=1}^{n}\frac{P(g_{i}|D^{+})}{P(g_{i}|D^{-})}$$ (5)

Additional details for calculating P(g_i_|D+) and P(g_i_|D-) are given in S1 File.

For any person with genotype G_i_, the 5 year risk of breast cancer can be estimated as:

$$P\left(D^{+}|G_{i}\right)=\;\frac{\frac{P{\left(D^{+}\right)}_{i}}{1-P{\left(D^{+}\right)}_{i}}LR_{Gi}}{\frac{P{\left(D^{+}\right)}_{i}}{1-P{\left(D^{+}\right)}_{i}}LR_{Gi}+1}$$ (6)

Where P(D^+^)_i_ is the 5-year risk probability projected by the BCSC model for the individual.

Since the relative risk associated with genotype γ is not directly measured in studies, we assume that γ is approximately equivalent to the odds ratio for breast cancer calculated from case-control studies, which is generally considered a good approximation when the prevalence of the disease is low. While the lifetime risk of disease is ~12%, the prevalence of disease in the general population remains <10%.

#### Modeling family history and SNPs

For family history, there is an expectation that the polygenic risk score will underlie some of the risk associated with family history. To correct for the effect of family history, we modified the calculation of risk by calculating the proportion of the risk associated with family history that is accounted for by the SNPs and accounting for that in the calculation of the posterior probability for women with a family history (see S1 File).

#### Assessing discrimination of the SNPs

To assess the discrimination of the SNPs together, we simulated genotypes for each of the SNPs based on the expected distribution of SNP in 5000 cases and 5000 controls (see equations in S1 File section A). We then calculated the likelihood ratios for each simulated case and control using the approach described above. We determined the receiver operating characteristic (ROC) area under the curve (AUC)

All simulations and calculations were performed using Stata Version 14.

## Results

### Distribution of Risk by BCSC Model

Approximately 54% of the women were age 50 or older, and 11.4% had a family history of breast cancer in at least one first degree relative (Table 1). The most common category of mammographic density was scattered fibroglandular (~42% of women) followed by heterogeneously dense (41%), extremely dense (10%) and almost entirely fatty (7.5%).

**Table 1:** Proportion of each risk group with >3% probability of breast cancer before and after SNP testing.

	% of population	% of risk group >3% Risk Pre-SNP with BCSC model alone	% of risk group >3% Risk with BCSC model and SNPs
All Women		6.8	9.6
Density			
Almost Entirely Fatty	7.5	0.4	1.2
Scattered Fibroglandular	41.8	2.3	6.4
Heterogeneously Dense	41.0	11.4	13.1
Extremely Dense	10.0	11.6	14.8
Ages			
35–49	46.0	0.2	1.5
50–69	54.0	12.2	16.7
Family History			
negative	73.3	4.5	7.6
positive	11.4	24.9	25.6
missing	15.4		

Of the entire screened population, 6.9% had a 5-year risk of ≥3% according to the BCSC model prior to consideration of genetic risk, qualifying them for consideration for chemoprevention from breast cancer (Table 1). The fraction of women above that risk threshold was <0.5% among women younger than age 50 and among women with almost entirely fatty breasts (Table 1). In contrast, ~11–12% of women with heterogeneously dense or extremely dense breasts are above the threshold for consideration of chemoprevention. Among women with family history of a first degree relative with breast cancer, approximately 25% of women exceed the threshold of 3% risk.

### Assessment of Polygenic Risk Discrimination

We evaluated the overall discrimination of the SNPs by simulating the SNPs in cases and controls (S1 Fig). To assess the discrimination of the SNPS, we calculated polygenic risk score in simulated cases and controls and determined the receiver operating characteristic (ROC) area under the curve (AUC) for predicting case vs. control status. The ROC AUC was approximately 0.63, a value that is consistent with previous studies[28, 33].

### Distribution of Risk Combining BCSC and Genotypes

**Fig 1:**
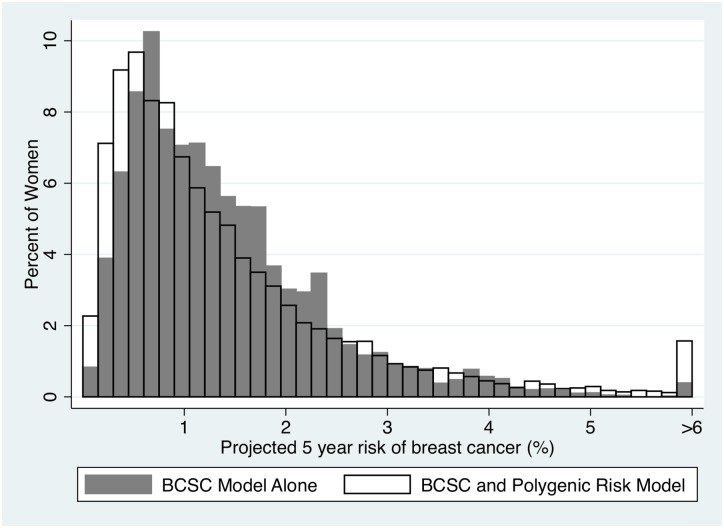
Distribution of risk in the BCSC pre- and post-genetic testing. The X-axis described the 5-year calculated probabilities for breast cancer. The Y-axis describes the fraction of the population at each interval of risk for women using just the BCSC model (yellow bars) and the BCSC model with SNPs (red bars.) The last vertical bar represents the fraction of the population with ≥ 6% risk of breast cancer in the next 5 years.

We evaluated the utility of adding SNPs to the BCSC model. Overall, adding risk information from the SNPs increased the variation in predicted risk, increasing the number of women at the lowest risk and the highest risk groups (Fig 1). At the threshold of chemoprevention, adding risk information from the SNPs increased the percentage of women who are candidates for chemoprevention from 6.9 to 9.6% (Table 1). However, the SNPs tend to have a greater impact on the highest category of risk (>4.0% risk) increasing the percentage of women in this group from 2.3% to 4.7% of the population (Table 2 and Fig 1). In addition, we found that the SNPs also reclassify a substantial proportion of women below the threshold for chemoprevention. Of the women who are classified by the BCSC model alone as ≥3% risk in 5 years, 40% (2.7% of 6.8%) are reclassified below that threshold after SNP testing (Table 2).

**Table 2:** Reclassification of risk with genetic testing. Percentages in each cell represent the percentage of the total population. Sections shaded in lighter gray and darker gray represent the group of women reclassified above and below the threshold for chemoprevention, respectively.

Pre-SNP Testing Risk	Post –SNP testing risk								
		<1.0%	1.0–1.4%	1.5–1.9%	2.0–2.4%	2.5–2.9%	3.0–3.9%	>4.0%	Total
	<1.0%	35.4%	4.5%	1.4%	0.3%	0.1%	0.1%	0.0%	41.7%
	1.0–1.4%	9.7%	6.1%	3.1%	1.4%	0.8%	0.6%	0.2%	21.8%
	1.5–1.9%	3.3%	4.6%	3.3%	2.1%	1.2%	0.7%	0.5%	15.7%
	2.0–2.4%	0.8%	1.9%	2.2%	1.6%	1.1%	1.1%	0.7%	9.4%
	2.5–2.9%	0.2%	0.6%	0.8%	0.7%	0.6%	0.9%	0.8%	4.6%
	3.0–3.9%	0.1%	0.2%	0.6%	0.6%	0.7%	1.0%	1.5%	4.6%
	>4.0%	0.0%	0.1%	0.2%	0.2%	0.3%	0.5%	1.1%	2.3%
	Total	49.4%	18.0%	11.4%	6.9%	4.7%	4.9%	4.7%	

For all categories of risk, more women were re-classified as above the threshold of ≥3% for chemoprevention after SNP testing (Table 1). Women with no family history of breast cancer and women with mammograms that had scattered fibroglandular tissue were the most likely to be reclassified above threshold for chemoprevention. Women with a positive family history were most likely to be above the threshold before testing and were least likely to be reclassified above the threshold after testing.

### Testing Strategies Using SNPs on Subsets of Women

**Fig 2:**
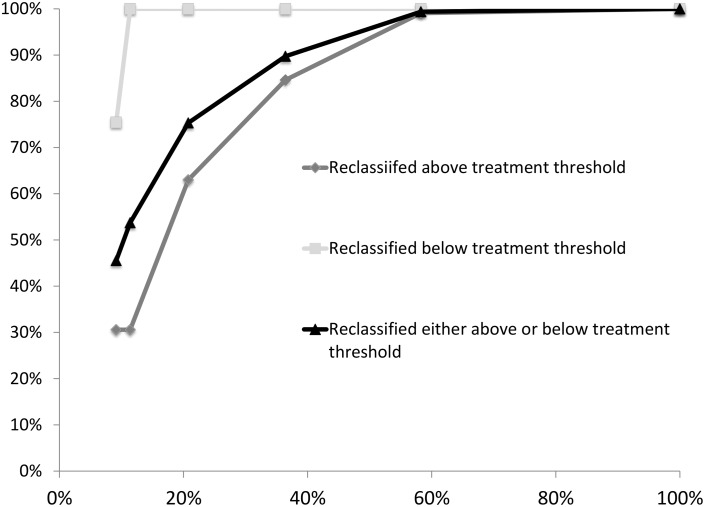
Percent of total benefit derived by SNP testing as a function of testing different percent of population. The X-axis represents the percent of the population tested in each scenario. The Y-axis represents the percent of the benefit derived from testing for reclassification of women. The values on the Y-axis are derived from each scenario in Table 3. Light gray lines with squares represents the percent of reclassification that occurs to below the treatment threshold out of the total that can be reclassified if everyone is tested. Medium gray line with diamonds represents the percent of reclassification that occurs to above the treatment threshold. The black gray line with triangles represents the percent of any reclassification (above or below the treatment threshold) out of the total possible reclassification if everyone were tested. For example in scenario 1, 9.2% of women are tested (X-axis) and 1.7% out of a possible 5.5% (30.6%) are reclassified above the treatment threshold (first medium diamond), 2.1% out of a possible 2.7% (75.5%) are reclassified below the treatment threshold (light gray square), and 3.8% out of a total possible 8.2% (45.5%) are reclassified either above or below the treatment threshold (black triangle).

We evaluated the application of SNP genotyping at different thresholds of risk to determine at what threshold testing may be most informative (Table 3). In general, testing women who are already above the 3% risk threshold was an efficient strategy since this group is relatively small and a large proportion of them are reclassified below the threshold for chemoprevention after SNP testing. Among women below the threshold, testing was most efficient for women near the threshold, as expected, while testing women who are far from the threshold (<1.0% BCSC model risk) yielded little additional benefit. Thus, strategies that test SNPs on a relatively modest fraction of women generally yield most of the benefit of reclassification. For example, a strategy that tests all women above a BCSC model calculated risk threshold of 2.0% 5-year risk (testing strategy 3, Table 3) would test 21% of women and yield 75% of the total reclassification from SNPs (Fig 2). A strategy that tests all women above a BCSC model calculated risk threshold of 1.5% 5-year risk (testing strategy 4, Table 3) would test 36% of women, yielding 90% of the total reclassification from SNPs (Fig 2). Finally, a testing strategy that includes all women with baseline risk of >1% would test ~58% of the entire population and yield 99% of all of the reclassification of testing the entire population.

**Table 3:** Strategies for adding genetic testing and yield of reclassification in terms of fraction of women tested and fraction of women reclassified.

Testing strategy	Range of Pre-test probabilities tested	Percent of population Tested	Percent who are reclassified to >3%	Percent who are reclassified to <3%
1	2.5–3.9% risk	9.2%	1.7%	2.1%
2	>2.5% risk	11.4%	1.7%	2.7%
3	>2% risk	20.8%	3.4%	2.7%
4	>1.5% risk	36.4%	4.6%	2.7%
5	>1% risk	58.3%	5.4%	2.7%
6	All	100.0%	5.5%	2.7%

## Discussion

We combined data from the BCSC with a modeling approach to examine the utility of common SNP testing for risk stratifying women in the context of making decisions about chemoprevention. We found that testing only a fraction of the women below the threshold for chemoprevention yields most of the benefit of testing all women below the threshold. In contrast, we found that testing all of the women above the threshold for chemoprevention is a useful strategy since a large fraction are reclassified below the threshold after SNP testing. Strategies that tested all women above 2.0% or above 1.5% BCSC model risk were relatively efficient and captured the majority of benefit of testing the entire population. However, the ultimate thresholds for testing will be determined by the cost of testing as well as by the potential other benefits of testing. For example, if the risk from SNPs can be used to make recommendations about age of onset of breast cancer screening and/or intensity of breast cancer screening or screening modality then SNP testing may be more useful in a larger number of women.

Previous studies have also used modeling approaches to combine the risk of traditional breast cancer risk factors and genetic information. Studies of risk prediction from the first 7 SNPs identified by GWAS suggested that the risk stratification with these SNPs alone was modest[34]. An empirical study of 10 SNPs found that the SNPs were insufficient for risk stratification in the general population[35]. Subsequently, Darabi et al showed that adding 18 SNPs demonstrated substantial additional risk stratification with SNPs when they are added to risk factors from the Gail model and to mammographic density[36]. Most recently, Brentnall et al. showed that addition of 67 SNPs from GWAS increased the distribution of risk in the population[37]. Garcia-Closas et al have modeled the combination of using non-genetic factors and SNPs to predict breast cancer risk, and used it to predict impact on screening and chemoprevention[38]. They used the UK NICE guidelines which recommend women lifetime risk >30% receive chemoprevention and women with a risk 17–30% as range to consider chemoprevention. These thresholds are difficult to directly compare to our analyses with 5 year risk; however we note that overall Garcia-Closas predicted ~10% of women would be recommended for chemoprevention which is very close to our results of 9.6%. Our study differs from from Garcia-Closas et al, since we also seek to define what fraction of the population would need to be tested to derive the benefits in terms of reclassification. Our study also differs from Garcia-Closas and other previous studies since we model women with a family history separately. We use an approach that models the expectation of increased frequency of risk alleles among women with a family history and also incorporates the empirically observed attenuation of family history after accounting for the polygenic risk score. So et al have developed a different approach to incorporating family history and SNPs by determining using an assumption that genetic risk follows a normal distribution in the population [39]. Such an approach would not require any empirical data about the attenuation of family history by SNPs. While the set of common risk SNPs only account for a modest fraction of the increased risk associated with family history, as more risk variants are identified, especially less common variants with a higher relative risk, the fraction of family history explained by these SNPs will increase and this correction will become more important.

Our approach was formulated around the question of chemoprevention for women with a 5 year risk of ≥3% based on the USPSTF recommendations. However, individual considerations for patients may alter that threshold. For example, for women who are older and at higher risk of thromboembolic disease, the threshold may be higher; conversely, younger women who are at lower risk for thromboembolic disease and uterine cancer, may have a better benefit to risk ratio at a lower breast cancer risk.

Our modeling made an assumption of independence of the SNPs. If there are substantial interactions between the SNPs, then this approach would not be accurate. In particular, if negative interactions occur between SNPs, then this approach would overestimate risk. Conversely, if some SNPs have positive interactions, this approach would underestimate risk. Previous studies which have empirically examined interactions between SNPs for breast cancer have not identified significant interactions after adjusting for multiple hypothesis testing[28, 29]. In order to assess how our model using an independence assumption across the unlinked SNPs works in comparison to real data, we simulated cases and controls and found a receiver operating characteristic (ROC) area under the curve (AUC) of 0.63. Previous reports have found an ROC AUC for the PRS in a range of 0.61[33] to 0.68[18]. The largest study by Mavaddat et al[28], with a dataset with over 33,000 cases and 33,000 controls, found an ROC AUC of 0.62 (95% CI: 0.62–0.63), consistent with our simulations. Furthermore, Mavaddat et al specifically tested for SNP by SNP interactions and ultimately chose to use the assumption of independence across SNPs as we did.

Our modeling approach cannot directly address the question of how much risk prediction improves with the addition of SNPs to traditional risk factors and mammographic density since empirical data is required to determine the overlap in model in risk prediction between SNPs and other risk factors. Vachon et al [18] have previously addressed this directly using the BCSC model and adding a polygenic risk score using 76 SNPs. They found that the polygenic risk was independent of the BCSC risk model and that adding the polygenic risk to the BCSC model improved the receiver operating characteristic area under the curve from ~0.66 to 0.69, a significant (p<0.001) improvement. They found that adding polygenic risk score to the BCSC model reclassified an additional ~10% of cases and ~10% of controls.

We used the common breast cancer risk variants that have been published to date and only considered SNPs that have genome-wide significant evidence. It is likely that other SNPs may be added to this list as more fine-mapping is done at the known loci and/or more GWAS is pursued. In addition, rare variants that cannot be detected by GWAS but are being found by sequencing studies could also be added to genetic testing panels for breast cancer risk. Additional risk variants should increase the predictive power of genetic testing and would likely change the thresholds for testing that we found. However, the general principles and modeling we used should still be applicable to defining new thresholds for testing.

We eliminated all SNPs with even modest linkage disequilibrium (D’>0.2) a conservative requirement. As a result, we used only 70 SNPs in our simulations, which is a bit fewer than other modeling efforts have used[38]. In practice, as fine mapping improves the causal variant/s at each locus, a more precise risk estimate can be achieved, by entering only causal variant/s at each locus and accounting for the linkage disequilibrium between them.

We used the assumption that the risk SNPs are independent of each other and independent of all other factors in the BCSC model except for family history. Several of these SNPs should not be independent of mammographic density, since they are known to increase density. However, the effect of these SNPs on mammographic density is very modest; to date less than 2% of the variance of mammographic density has been explained[40]; therefore, in practice density and the SNPs included in our study can be considered nearly independent consistent with recent observations where a model using density was independent of polygenic risk[18].

We focused on breast cancer risk in Caucasian women since the information about SNPs is based on far larger sample sizes for both discovery and replication compared to other populations. Our analyses should be repeated for other racial/ethnic groups as the SNP and rare variant lists in these populations is improved.

Our analyses focused on the risk of all invasive breast cancer since our approach was centered around the USPSTF recommendations, which are based on the risk for all invasive breast cancer. Therefore, we used BCSC 5-year risk, which is based on the risk of all invasive breast cancer and we used the SNP risk for any invasive breast cancer, rather than risk for specific breast cancer subtypes. However, the current chemoprevention agents reduce estrogen receptor (ER)-positive breast cancer but do not affect ER-negative breast cancer risk. In principle, the risks and benefits and risk threshold for chemoprevention could be modified to focus on ER-positive breast cancer risk. In addition, breast cancer risk models and SNP based risk stratification could also be focused on ER-positive breast cancer. However, since most breast tumors are ER-positive, SNP-based models designed for overall breast cancer perform well for ER-positive breast cancer[41]. Another assumption behind our approach is that the women who are identified as high risk by SNPs will be protected from breast cancer by chemoprevention to the same degree that women who were included in the breast cancer prevention trials. Recent data from breast cancer prevention trials re-analyzed to include SNP-based risk, suggests that SNPs can be used to find women who would benefit from chemoprevention with SERMs[41]. If women identified as high risk based on SNPs have an intrinsically different response to preventive treatments, the risk/benefits would need to be reconsidered.

The decision of whether to use a risk model and to add genetic information to the model in the context of decision making about preventive therapy depends on each woman’s perception of the benefits and harms of therapy. Currently, prevention with tamoxifen or other endocrine therapies remains low. A survey of health records in 2010 estimated that over 20,000 women are taking tamoxifen for prevention in the U.S. and nearly 100,000 post-menopausal women are taking raloxifene, but noted this is a very small fraction of the women estimated to potentially benefit[4]. However, willingness to consider tamoxifen may be considerably higher with studies finding that 40–50% of women who are at risk may be willing to take the medication [42]. Thus, the low uptake may, at least in part, be due to physician lack of awareness or willingness to prescribe endocrine therapies in the preventive setting. Improved risk stratification with SNPs should lead to better selection of patients for chemoprevention and could increase physicians’ willingness to prescribe preventive therapies[43].

Risk stratification with SNPs and other genetic variants may also be useful to aid in other decisions for breast cancer prevention and screening. For example, in deciding whether to initiate mammography at age 40 or age 50. The thresholds for SNP testing we developed in this paper are only useful in the context of the USPSTF guidelines for chemoprevention; however, the approach we describe could also be used to define a testing approach with SNPs for when to initiate mammography screening and whether to use MRI using the risk threshold for those interventions.

In summary, we found that testing ~21–36% of women for genetic variants would derive the vast majority of benefit in terms of identifying women for primary breast cancer risk prevention. As information on genomic risk factors becomes more readily available, individualized risk assessment integrating the genomic and non-genomic risk information for patients and clinicians should enhance risk counseling and medical decision making.

## Supporting Information

S1 Fig The distribution of polygenic risk scores in cases and controls. This histogram represents the distribution of the log (base 10) of the likelihood ratios of the polygenic risk scores in 5000 simulated breast cancer cases and 5000 simulated women without breast cancer. Each case was simulated using the genotype probabilities described for breast cancer cases and for unaffected women from section A of S1 File. The likelihood ratio was then calculated as described in the methods section. We also calculated the ROC AUC for the likelihood ratio for breast cancer cases vs. unaffected women, finding an AUC of 0.63.
(PDF)

S1 File Supplementary Methods. This file includes details of the modeling. Section A includes all of the equations for each of the conditional probabilities for each genotype used in the calculation of the likelihood ratios described in the method section. Section B includes details of the modeling of the SNPs conditional on family history.
(DOCX)

S1 Table The distribution of Pre-genotyping risk. The distribution of the percentage of women in each 5 year risk category is displayed. The 5 year risk was calculated in 796,294 women undergoing screening mammography using the Breast Cancer Surveillance Consortium. The risk distribution is displayed separately for all women (first column) and for subsets of the population by different risk factors. These risk distributions were used to simulate the pre- genotyping risk scores.
(XLSX)

S2 Table Single nucleotide polymorphisms list. This list includes the final list of 70 SNPs used in the modeling.
(XLSX)
