# Supplementary figures and images for: Using Breast Cancer Risk Associated Polymorphisms to Identify Women for Breast Cancer Chemoprevention
Source: PLoS One. 2017 Jan 20;12(1):e0168601. doi: 10.1371/journal.pone.0168601 (PMC5249071; doi:10.1371/journal.pone.0168601)

Supplementary Figure 1

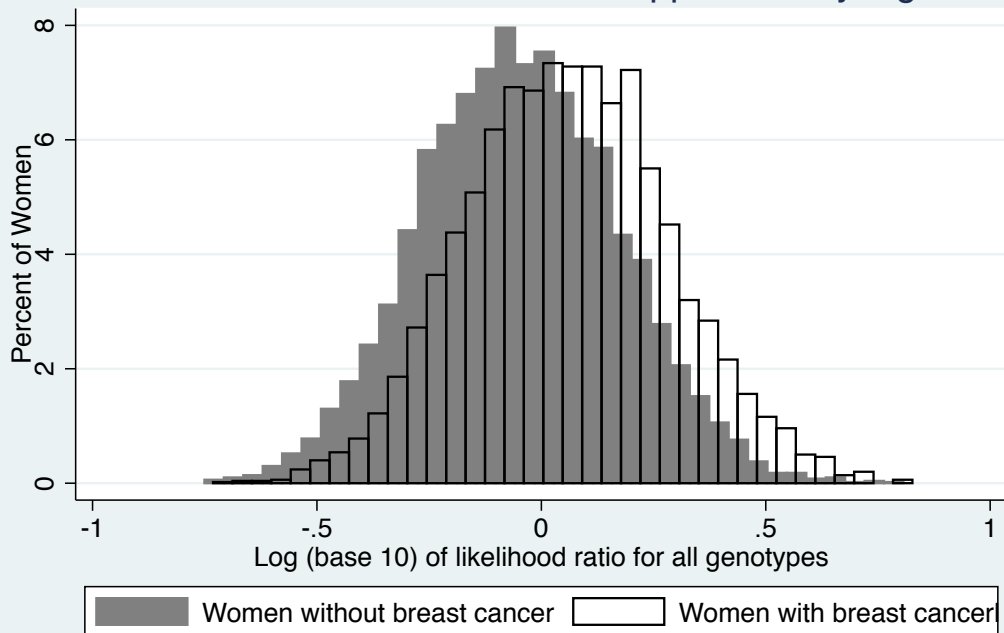

ROC AUC for breast cancer cases vs. unaffected women = 0.63

Supplement: S1 Fig — This histogram represents the distribution of the log (base 10) of the likelihood ratios of the polygenic risk scores in 5000 simulated breast cancer cases and 5000 simulated women without breast cancer. Each case was simulated using the genotype probabilities described for breast cancer cases and for unaffected women from section A of S1 File. The likelihood ratio was then calculated as described in the methods section. We also calculated the ROC AUC for the likelihood ratio for breast cancer cases vs. unaffected women, finding an AUC of 0.63. (PDF) [file pone.0168601.s001.pdf]
