# Supplementary material for: Using Breast Cancer Risk Associated Polymorphisms to Identify Women for Breast Cancer Chemoprevention
Source: PLoS One. 2017 Jan 20;12(1):e0168601. doi: 10.1371/journal.pone.0168601 (PMC5249071; doi:10.1371/journal.pone.0168601)
Supplement: S1 File — This file includes details of the modeling. Section A includes all of the equations for each of the conditional probabilities for each genotype used in the calculation of the likelihood ratios described in the method section. Section B includes details of the modeling of the SNPs conditional on family history. (DOCX) [file pone.0168601.s002.docx]

**Supplementary Materials:**

**Supplementary Methods:**

A. Risk for individual genotypes:

For a biallelic marker with alleles A, a, with frequencies p and 1-p, respectively with genetic risk γ per each additional A allele, the probability of the genotypes given affection status are given as:

1.

$$P\left( AA | D^{+} \right)=\frac{p^{2}\gamma^{2}}{p^{2}\gamma^{2}+2p\left( 1-p \right)\gamma+{(1-p)}^{2}}$$

2.

$$P\left( Aa | D^{+} \right)=\frac{2p\left( 1-p \right)\gamma}{p^{2}\gamma^{2}+2p\left( 1-p \right)+{(1-p)}^{2}}$$

3.

$$P\left( aa | D^{+} \right)=\frac{{(1-p)}^{2}}{p^{2}\gamma^{2}+2p\left( 1-p \right)+{(1-p)}^{2}}$$

$$P\left( AA | D^{-} \right)={\frac{p}{1-K}}^{2}-\frac{p^{2}\gamma^{2}K}{{(1-K)(p}^{2}\gamma^{2}+2p\left( 1-p \right)\gamma+\left( 1-p \right)^{2})}$$

$$P\left( Aa | D^{-} \right)={\frac{2p(1-p)}{1-K}}-\frac{2p(1-p)K}{{(1-K)(p}^{2}\gamma^{2}+2p\left( 1-p \right)\gamma+\left( 1-p \right)^{2})}$$

$$P\left( aa | D^{-} \right)={\frac{(1-p)}{1-K}}^{2}-\frac{{(1-p)}^{2}K}{{(1-K)(p}^{2}\gamma^{2}+2p\left( 1-p \right)\gamma+\left( 1-p \right)^{2})}$$

Thus,

$${LR}_{AA}= \frac{P(AA|D^{+})}{PAA|D^{-})}$$

$${LR}_{Aa}= \frac{P(Aa|D^{+})}{PAa|D^{-})}$$

$${LR}_{aa}= \frac{P(aa|D^{+})}{P(aa|D^{-})}$$

B. Simulation of genotypes according to family history:

For the purposes of this simulation we pay attention to first degree relatives (affected mothers or sisters). Given an individual with a family history, their affected mother’s or sister’s genotype would be given by equations for P(AA|D+), P(Aa|D+) and P(aa|D+) given above.

The genotype of the individual is then a function of the relatedness and the individual. For siblings at autosomal loci the genotypic relatedness is:

|  | Sister 1 (Known genotype) | | | |
| --- | --- | --- | --- | --- |
| Sister 2 (proband) |  | AA | Aa | aa |
|  | AA | ¼+ ½ p+ ¼ p^2^ | ¼ p + ¼ p^2^ | ¼ p^2^ |
|  | Aa | ½ (1-p) + ½ p (1-p) | ¼ + ¼ p + ¼ (1-p) + ½ p(1-p) | ½ p + ½ p (1-p) |
|  | aa | ¼ (1-p)^2^ | ¼ (1-p) + ¼ (1-p)^2^ | ¼ + ½ (1-p) + ¼ (1-p)^2^ |

For mother-daughter pairs the genotypic relatedness

|  | Mother | | | |
| --- | --- | --- | --- | --- |
| Daughter (proband) |  | AA | Aa | aa |
|  | AA | p | ½ p | 0 |
|  | Aa | 1-p | ½ p + ½ (1-p) | p |
|  | aa | 0 | ½ (1-p) | 1-p |

For a woman with an affected sister the probabilities of each genotype are:

$$P\left( AA | {sister D}^{+} \right)=P\left( AA | D^{+} \right)\left( \frac{1}{4}+\frac{1}{2}p+\frac{1}{4}p^{2} \right)+P\left( Aa | D^{+} \right)\left( \frac{1}{4}p+\frac{1}{4}p^{2} \right)+P\left( aa | D^{+} \right)\frac{1}{4}p^{2}$$

$$P\left( Aa | {sister D}^{+} \right)=P\left( AA | D^{+} \right)(\frac{1}{2}\left( 1-p \right)+\frac{1}{2}p(1-p)+P\left( Aa | D^{+} \right)\left( \frac{1}{4}+\frac{1}{4}p+ \frac{1}{4}\left( 1-p \right)+\frac{1}{2}p\left( 1-p \right) \right)+P\left( aa | D^{+} \right)(\frac{1}{2}p+\frac{1}{2}p\left( 1-p \right))$$

$$P\left( aa | {sister D}^{+} \right)=P\left( AA | D^{+} \right)\left( \frac{1}{4}(1-{p)}^{2} \right)+P\left( Aa | D^{+} \right)\left( \frac{1}{4}(1-p)+\frac{1}{4}(1-{p)}^{2} \right)+P\left( aa | D^{+} \right)(\frac{1}{4}{+\frac{1}{2}\left( 1-p \right)+\frac{1}{4}\left( 1-p \right)}^{2})$$

For a woman with an affected mother the probabilities for each genotype are:

$$P\left( AA | {mother D}^{+} \right)=P\left( AA | D^{+} \right)p+P\left( Aa | D^{+} \right)\frac{1}{2}p$$

$$P\left( Aa | {mother D}^{+} \right)=P\left( AA | D^{+} \right)\left( 1-p \right)+P\left( Aa | D^{+} \right)\left( \frac{1}{2}\left( 1-p \right)+ \frac{1}{2} P \right)+P\left( aa | D^{+} \right)p$$

$$P\left( aa | {mother D}^{+} \right)=P\left( Aa | D^{+} \right)\left( \frac{1}{2}(1-p) \right)+P\left( aa | D^{+} \right)(1-p)$$

For the simulations of genotypes, we assumed that ½ of the women with family history had an affected sister and half had an affected mother. We did not explicitly model the proportion of women with both an affected sister and mother since the BCSC model does not distinguish these women.

For women without a family history, we assumed that the genotype frequencies were approximately the same as the distribution of genotypes in the general population.

Since part of the effect of family history is accounted for by the SNPs, then the increased risk associated with family history based on the BCSC model needs to be adjusted for the SNPs. Ideally, we would have access a large dataset that combines the BCSC risk factors including family history and the SNPs we have modeled. Since such a dataset is not currently available, we use recently published data by Mavaddat et al [39] to make the adjustment. We note that the odds ratio from family history is ~1.81 without adjustment for SNPs and ~1.68 with adjustment for SNPs. Thus, the correction for odds ratio that incorporates both family history and SNPs is ~1.68/1.81 or 0.93. Since the BCSC risk model used the odds ratio for family history as part of the model, this correction should be approximately correct in the BCSC risk population. Thus, for women with a family history, we multiplied the BCSC risk score by 0.93 prior to applying the algorithm described above for polygenic risk score.
